# The Confluence of Stereotactic Ablative Radiotherapy and Tumor Immunology

**DOI:** 10.1155/2011/439752

**Published:** 2011-11-15

**Authors:** Steven Eric Finkelstein, Robert Timmerman, William H. McBride, Dörthe Schaue, Sarah E. Hoffe, Constantine A. Mantz, George D. Wilson

**Affiliations:** ^1^21st Century Oncology Translational Research Consortium, Scottsdale, AZ 85251, USA; ^2^Department of Radiation Oncology and Neurologic Surgery, University of Texas Southwestern Medical Center, Dallas, TX 75391, USA; ^3^Department of Radiation Oncology, David Geffen School of Medicine at UCLA, Los Angeles, CA 90095, USA; ^4^Department of Radiation Oncology, Moffitt International, Tampa, FL 33612, USA; ^5^Division of Radiation Biology, William Beaumont Hospital, Royal Oak, MI 48073, USA

## Abstract

Stereotactic radiation approaches are gaining more popularity for the treatment of intracranial as well as extracranial tumors in organs such as the liver and lung. Technology, rather than biology, is driving the rapid adoption of stereotactic body radiation therapy (SBRT), also known as stereotactic ablative radiotherapy (SABR), in the clinic due to advances in precise positioning and targeting. Dramatic improvements in tumor control have been demonstrated; however, our knowledge of normal tissue biology response mechanisms to large fraction sizes is lacking. Herein, we will discuss how SABR can induce cellular expression of MHC I, adhesion molecules, costimulatory molecules, heat shock proteins, inflammatory mediators, immunomodulatory cytokines, and death receptors to enhance antitumor immune responses.

## 1. Introduction

Stereotactic radiosurgery (SRS) was originally developed for the treatment of intracranial tumors and has demonstrated clinical effectiveness in treating a variety of benign and malignant conditions. Its extracranial counterpart, stereotactic body radiation therapy (SBRT), also known as stereotactic ablative radiotherapy (SABR), has more recently shown efficacy for the treatment of tumors in organs such as the liver and lung. The potential for using SABR is likely greater than for SRS given the larger volume of potential indications outside the central nervous system. Technology, rather than biology, is driving the rapid adoption of SABR in the clinic due to advances in precise positioning, motion control, dosimetry, and precise targeting with image guidance. Dramatic improvements in tumor control have been demonstrated in several studies due to the demonstration that very potent dose can be delivered by use of the mentioned technology. However, our knowledge of normal tissue biology response mechanisms to large fraction sizes is relatively lacking compared to conventional fractionation.

## 2. Radiobiologic Considerations

A fundamental issue in SABR is whether classical radiobiologic modeling with the linear-quadratic (LQ) model is a valid method to assess the biologically effective dose at the high doses typically encountered in radiosurgery. This point was debated in back-to-back papers in seminars in radiation oncology [1, 2], where Brenner argued that LQ formalism was appropriate whilst Kirkpatrick and colleagues suggested it was inappropriate. Brenner's argument is based on the robustness of the LQ model to predict fractionation and dose-rate effects in experimental models *in vitro* and *in vivo* at doses up to 10 Gy. This conclusion is based on the premise that cell killing is the dominant process mediating the radiotherapeutic response for both early and late effects including vascular effects. Brenner argued that, to date, there is no evidence of problems when LQ has been applied in the clinic. 

However, this was the crux of Kirkpatrick and colleagues' argument. They noted multiple studies demonstrating that the administration of a single high dose of radiation *in vivo* had a much greater effect than predicted by the LQ model; they cited several examples including Leith et al. [3] who calculated that the dose to obtain a high probability of tumor control for brain lesions would be at least 25 to 35 Gy using the LQ model, which was much higher than the observed clinically effective radiosurgical dose, which was in the range of 15–20 Gy. Kirkpatrick maintained that there was a disconnect between *in vitro* cell survival data and observed clinical data which suggests that there is more than one mechanism of radiation damage and that these operate differentially at low and high doses. In addition, Kirkpatrick argues that the LQ model does not effectively address the potential existence of radioresistant cancer stem cells, which may require a threshold dose to be crossed before their death is triggered. 

Several authors have proposed alternate models to the LQ. In all cases, they argue that the LQ model was intended as a low dose mathematical representation of the data constituting the survival curve [4, 5]. As most survival curves demonstrate a curvilinear “shoulder” followed by a linear portion on a linear log scale, the high-dose trend to endlessly curve associated with the LQ model overpredicts survival at high dose per fraction from a purely mathematical perspective. In the case of the universal survival curve of Park et al. [5], the strength of the LQ in the low dose realm is exploited but abandoned for the linear multitarget model in the high-dose realm. Thus, the *in vitro* survival curve has goodness of fit in all clinically significant ranges including the ablative range characteristic of SABR. Admittedly, none of the proposed mathematical models properly account for *in vivo* effects including vascular and immune contribution to cell death.

## 3. The Role of Tumor Stroma

As stated above, the accepted rationale for radiotherapy (RT) is based on causing lethal DNA damage to tumor cells and the tumor-associated stroma. There is unequivocal evidence which has been presented by Fuks and colleagues that the tumor stroma plays an important role in the response to high dose per fraction radiation treatment. They demonstrated that vascular endothelial cell apoptosis is rapidly activated above 10 Gy per fraction [6], and that the ceramide pathway orchestrated by acid sphingomyelinase (ASMase) operates as a rheostat that regulates the balance between endothelial survival and death and thus tumor response [7]. These studies relied heavily on mice that had ASMase knocked out in all tissues; the authors have countered the argument that defective immune system that is known to occur in ASMase −/− mice [8] influenced their observations [6]. 

Damage to vascular/stromal elements in tumors has also been observed around 2 weeks after radiation exposure that was less dependent on size of dose per fraction [9]. Pathological observations show profound changes in vasculature after radiosurgery and from studies on arteriovenous malformations [10], where obliteration of abnormal vasculature occurs months after irradiation, but is rarely seen below single doses of 12 Gy climbing steeply with increasing doses above this threshold.

In terms of the infiltrating immune cell component of tumor stroma, conventional RT has traditionally been viewed as immunosuppressive [11], but the systemic effects of both cancer and local radiotherapy of cancer on the immune system are clearly more complex than this. Although lymphocyte radiosensitivity is well recognized, the effects of different doses and delivery methods on systemic and locoregional naive, effector, or regulatory T cell or other immunologically relevant populations is still the subject of debate [12, 13]. Several authors have investigated the potential immunomodulatory effects of localized RT on tumors resulting in conflicting reports as to whether these responses promote or interfere with tumor reduction [14–16]. This dualism is something that is to be expected and is inherent in a system that has to promote both destruction of pathogens and tissue healing while regulating anti-self reactivity. It is also possible that the more positive effects seen in colorectal cancer where the immune score was significantly associated with differences in disease-free, disease-specific, and overall survival [17] are in part a reflection of additional microbial challenges that may not be present in other sites.

## 4. Direct Interaction between Radiation, Tumor Cells, and the Immune System

**Figure 1:**
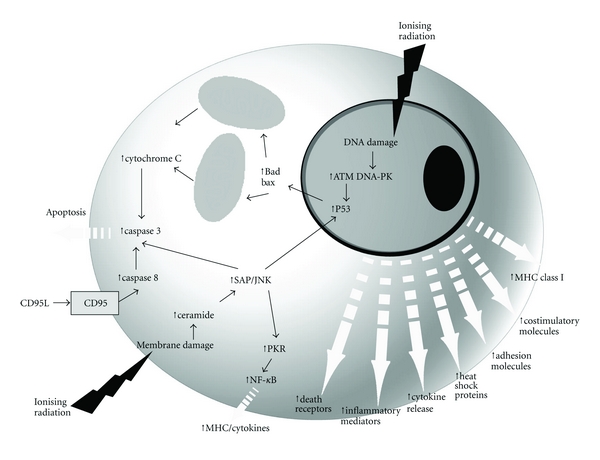
*Confluence of SABR and Immunotherapy.* Apoptosis can be initiated by SABR-induced DNA damage and upregulation of the p53 tumor suppressor gene. In addition, apoptosis can be triggered by SABR-induced damage to the cellular lipid membrane, which can induce ceramide formation and activate the SAPK/JNK signaling pathway. Thus, SAPK/JNK can upregulate PKR expression, which can induce MHC and cytokines via NF-*κ*B. SABR can induce cellular expression of MHC Class I, adhesion molecules, costimulatory molecules, heat shock proteins, inflammatory mediators, immunomodulatory cytokines, and death receptors.

Several lines of evidence have suggested direct immune modulation of immune cells by RT [15, 18–22]. Apetoh et al. showed that radiation can trigger signals that stimulate toll-like receptor 4 on antigen presenting dendritic cells (DCs) [18], Liao has shown that irradiation of DC can enhance presentation of antigenic peptides by the exogenous pathway and is a maturation signal, while inhibiting internal antigen processing [21], and Merrick has shown a decrease in IL-12 production that has a negative effect on presentation [15]. Several reports have shown increased expression of MHC class I and coaccessory molecules after radiation of both tumor and host cells, while Chakraborty et al. [19] reported a direct effect of radiation on tumors by modifying the phenotype of tumor cells to render them more susceptible to vaccine-mediated T-cell killing, and others have shown that radiation-induced changes in the tumor immune microenvironment to promotes greater infiltration of immune effector cells [22] (Figure 1).

## 5. Mechanisms of Radiation Driven Tumor Immunology

The early report of Stone [23] that the immune system can dramatically alter the dose required to obtain local tumor control has been updated by Lee and colleagues, who showed that CD8+ T cells could be responsible for the therapeutic effects of ablative radiation [24]. The delivery of an ablative dose of radiation of 15–25 Gy was found to cause a significant increase in T-cell priming in draining lymphoid tissue, leading to reduction or eradication of the primary tumor or distant metastasis in a CD8+ T-cell dependent fashion in an animal model. While conventional 2 Gy doses seem inferior at generating such responses, higher sized dose fractions may be better than single doses [25].

The possibility that there may be a certain dose per fraction that is optimal for stimulating radiation adjuvanticity is of relevance to mechanism of radiation-induced immune stimulation and clinical practice. Conventional RT has already been shown to enhance tumor-specific T-cell responses [26], but such responses are likely of little clinical relevance and surely can be improved upon by optimizing dose delivery and integrating RT with modern immunotherapeutic strategies. 

Radiation can not only kill tumor cells releasing tumor antigens and molecules with what are collectively called damage-associated molecular patterns (DAMPs) that exert various immunomodulatory effects including induction of the expression of cytokines, chemokines, and release of inflammatory mediators [27–30] (Figure 1). Although proinflammatory cytokines generally are produced by higher doses than are conventionally used in RT, there may be an accumulating effect [31]. Radiation also increases the permeability of the local vasculature either directly or through cytokine production that leads to recruitment of circulating leukocytes into surrounding tissues including antigen-presenting cells and effector T cells [32–34]. Thus, a radiation-induced proinflammatory microenvironment within irradiated tumors could provide DCs with maturation inducing stimuli critical for eliciting effective antigen presentation. The obverse of this is that radiation can stimulate production of suppressor myeloid cells [35] and Treg cells [36] in a dose-dependent manner that presumably aim to dampen and contain tissue damage and that can be highly immunosuppressive. Thus, to “unmask” the more positive aspects of radiation killing on immunity, it may be necessary to target and impair these natural defenses.

Advances in the understanding of the mechanisms that regulate the development of antitumor immunity, as well as improved knowledge of the complex effects of radiation on tissues [37], have revived interest in the possibility of combining radiation and immune-based therapies to achieve a better local and systemic tumor control [28–31]. Since William Coley started treating patients at the end of the 19th century with bacterial toxins, there have been waves of enthusiasm promoting immunotherapy for the treatment of cancer. The introduction of cytokines, in particular interleukin-2 (IL-2), for cancer treatment was a major clinical effort that had modest success. Until recently, however, these efforts have been hampered by a lack of molecular definition of tumor antigens, a means of delivering them effectively, and a sensitive and reliable way to measure responses. 

This situation changed with the molecular cloning of human tumor-associated antigens that could be recognized by T cells, the ability to culture powerful antigen presenting cells (APCs) in the form of dendritic cells (DCs), and to assess immune responses to specific tumor epitopes using tetramer and ELISPOT assays [38]. These advances allied to the development of genetically modified mouse models have led to a deeper understanding of the interactions between cancer and the immune system of the host [39]. Indeed, the available experimental evidence supports the hypothesis that once tumors have become clinically apparent their immunogenicity has been modified by the selective pressure of the immune system, resulting in the growth of tumors that are characteristically poorly immunogenic, being able to escape immune detection, and/or to actively inhibit immune effectors [39]. Furthermore, it is clear that, although T cells become tolerant to many self-antigens in the thymus, which depletes the pool that might react to cancer, tolerance to many self-components is actively maintained in the periphery by several mechanisms. For example, immature DC presenting self-antigens to T cells are tolerogenic and peripheral tolerance is maintained by Tregs subset that can be innate or induced. Suppressor macrophages form a final barrier to immune function and can result in immune shutdown [40]. Peripheral tolerance can be broken by “maturation” of DC in local sites that allow transient immune responses to invading pathogens, but it leads to the belief that if it were not for these regulatory mechanisms T cells could respond better to “self-” antigens on tumors, something for which there is now considerable evidence [41]. 

The recognition of the fact that the host can break a state of tolerance that has developed to its own tumor offers many possibly effective immunotherapeutic strategies, some being currently tested in clinical trials. The “danger” model of immunity suggests that pathogens with associated molecular patterns (PAMPs) and DAMPS engender an inflammatory milieu that promotes the development of antigen-specific immunity through DC maturation that allows internalization of apoptotic and necrotic cellular debris and presentation of processed antigen to T cells. Thus, administration of radiation may therefore be considered to create an inflammatory setting via DC maturation, induction of apoptosis, necrosis, cell surface molecules, and secretory molecules. As with many other challenges, radiation upregulates expression of immunomodulatory surface molecules (MHC, costimulatory molecules, adhesion molecules, death receptors, heat shock proteins) and secretory molecules (cytokines, inflammatory mediators) in tumor, stromal, and vascular endothelial cells. Important amongst these may be the upregulation of TNF family members that could promote cell killing, not only by TNF in the microenvironment but also by radiation-induced TNF.

## 6. Can Radiobiologic Models Be Adapted to Account for Other Modes of Tumor Response at High Dose Per Fraction?

Therefore, the evidence would seem to suggest that there are several potential immunologic mechanisms for cell killing in the high-dose range. The LQ model has long been considered to overestimate radiation cell killing at these doses as a consequence of the model's prediction of a continuous downward bend (ßd2) in the survival curve. While *in vivo* data are sparse, the dose-response may be linear above 12 Gy [42], and two-component or other models have been described that may better predict the response at dose per fraction above 5–7 Gy. For example, Park et al. [5] described the effects of radiation in the ablative dose range using a universal survival curve (USC) model, which combines the LQ and multitarget models using a transition dose to separate the two fitting components of the model. Using the LQ model, the potency of the doses used in the Indiana University phase II trial of SABR for medically inoperable NSCLC (20 Gy × 3) was estimated to be 1.7 times greater than the biological effectiveness of a similar Japanese trial delivering 12 Gy × 4. However, when the USC model was used, the potency of the Indiana University regimen was only 1.34 times more than the Japanese regimen [5]. Other models have included the generalized LQ (gLQ) model in which the reduction of conversion of sublethal to lethal injury in hypofractionated ablative dose radiation is taken into account and the actual effect of the radiation is lower than what was estimated by the LQ model [43]. However, modeling may never fully describe the complexity of the biological processes involved in the response to high dose per fraction radiation, but it might facilitate the ability to design optimal radiosurgery treatment plans once sufficient clinical data have been obtained. From a radiobiological perspective, what is clear is that there are processes that are different at high from low dose per fraction and these include the ability of cells to progress through the cell cycle, the likelihood of cell death perhaps with a different mechanism, vascular effects, proinflammatory effects, and immune effects.

## 7. Local Radiation Enhancement of Systemic Immunity

It is clear from what has been said that localized cancer has systemic immune effects as does RT. It is also clear that the outcome of cancer and cancer therapy depends heavily upon the nature of the cells that are generated, in particular with respect to metastasis and overall survival. It seems likely that unexpected discrepancies in the relative efficacies of different anticancer regimens and divergence or convergence between regional and distant failures could be due to such systemic influences, for example, of local tumor control on the incidence of distance metastasis. Future studies aimed at assessing the predictive value of systemic responses in the response of cancer to different dose schedules of RT are likely to be very informative, and strategies that target systemic innate and cancer and radiation-induced regulatory mechanism hold great promise. These strategies, together with DC-based and other forms of antitumor vaccination, can greatly modify the total radiation dose required to achieve local control as well as influencing distant disease, and RT should adapt to optimally integrate with such approaches. While most chemotherapy regimens are thought to compromise the immune system, they also can have immunomodulatory effects that require study.

## 8. Conclusions

Searching for references on PubMed that contain “SBRT” or “SABR” and “biology” reveals very few hits emphasizing that this is an area of modern radiotherapy where detailed understanding biology needs to catch up with the clinic [44]. Small animal platforms are now developed to simulate a realistic SABR delivery in experimental animals [45] and other recent developments in image-guided small animal irradiators could also be adapted to simulate SABR [46]. A wealth of knowledge already exists in the radiobiology archive from the ‘60s, ‘70s, and ‘80s where large doses per fraction were used for ease of experimental design in experimental studies, which needs to be revisited. In the meantime, combination immunotherapy and radiation approaches are being translated into the clinic [47]. Currently, combination immunotherapy and radiation approaches are being translated into the clinic where intratumoral dendritic cell injection with coordinated irradiation and introduction of autologous, unmanipulated dendritic cells has been the subject of sarcoma therapy [48].

At present, SABR represents an exciting, effective, yet empirically designed radiation therapy. Increasing our knowledge of the underlying biology associated with modern high-dose delivery will only serve to improve the therapeutic benefit of this modality. In addition, we believe that SABR could be optimized for use with immunotherapeutic approaches so as to better generate tumor antigen-specific cellular immunity.

## References

[1] Brenner DJ (2008). The linear-quadratic model is an appropriate methodology for determining isoeffective doses at large doses per fraction. *Seminars in Radiation Oncology*.

[2] Kirkpatrick JP, Meyer JJ, Marks LB (2008). The linear-quadratic model is inappropriate to model high dose per fraction effects in radiosurgery. *Seminars in Radiation Oncology*.

[3] Leith JT, Cook S, Chougule P (1994). Intrinsic and extrinsic characteristics of human tumors relevant to radiosurgery: comparative cellular radiosensitivity and hypoxic percentages. *Acta Neurochirurgica, Supplement*.

[4] Guerrero M, Li XA (2004). Extending the linear-quadratic model for large fraction doses pertinent to stereotactic radiotherapy. *Physics in Medicine and Biology*.

[5] Park C, Papiez L, Zhang S, Story M, Timmerman RD (2008). Universal survival curve and single fraction equivalent dose: useful tools in understanding potency of ablative radiotherapy. *International Journal of Radiation Oncology Biology Physics*.

[6] Garcia-Barros M, Paris F, Cordon-Cardo C (2003). Tumor response to radiotherapy regulated by endothelial cell apoptosis. *Science*.

[7] Truman JP, García-Barros M, Kaag M (2010). Endothelial membrane remodeling is obligate for anti-angiogenic radiosensitization during tumor radiosurgery. *PLoS One*.

[8] Utermöhlen O, Karow U, Löhler J, Krönke M (2003). Severe impairment in early host defense against Listeria monocytogenes in mice deficient in acid sphingomyelinase. *Journal of Immunology*.

[9] Chen FH, Chiang CS, Wang CC (2009). Radiotherapy decreases vascular density and causes hypoxia with macrophage aggregation in TRAMP-C1 prostate tumors. *Clinical Cancer Research*.

[10] Szeifert GT, Kondziolka D, Atteberry DS (2007). Radiosurgical pathology of brain tumors: metastases, schwannomas, meningiomas, astrocytomas, hemangioblastomas. *Progress in neurological surgery*.

[11] Wasserman J, Blomgren H, Rotstein S, Petrini B, Hammarstrom S (1989). Immunosuppression in irradiated breast cancer patients: in vitro effect of cyclooxygenase inhibitors. *Bulletin of the New York Academy of Medicine*.

[12] North RJ (1986). Radiation-induced, immunologically mediated regression of an established tumor as an example of successful therapeutic immunomanipulation. Preferential elimination of suppressor T cells allows sustained production of effector T cells. *Journal of Experimental Medicine*.

[13] Demaria S, Kawashima N, Yang AM (2005). Immune-mediated inhibition of metastases after treatment with local radiation and CTLA-4 blockade in a mouse model of breast cancer. *Clinical Cancer Research*.

[14] Ohuchida K, Mizumoto K, Murakami M (2004). Radiation to stromal fibroblasts increases invasiveness of pancreatic cancer cells through tumor-stromal interactions. *Cancer Research*.

[15] Merrick A, Errington F, Milward K (2005). Immunosuppressive effects of radiation on human dendritic cells: reduced IL-12 production on activation and impairment of naïve T-cell priming. *British Journal of Cancer*.

[16] Reits EA, Hodge JW, Herberts CA (2006). Radiation modulates the peptide repertoire, enhances MHC class I expression, and induces successful antitumor immunotherapy. *Journal of Experimental Medicine*.

[17] Galon J, Costes A, Sanchez-Cabo F (2006). Type, density, and location of immune cells within human colorectal tumors predict clinical outcome. *Science*.

[18] Apetoh L, Ghiringhelli F, Tesniere A (2007). Toll-like receptor 4-dependent contribution of the immune system to anticancer chemotherapy and radiotherapy. *Nature Medicine*.

[19] Chakraborty M, Abrams SI, Coleman CN, Camphausen K, Schlom J, Hodge JW (2004). External beam radiation of tumors alters phenotype of tumor cells to render them susceptible to vaccine-mediated T-cell killing. *Cancer Research*.

[20] Finkelstein SE, Heimann DM, Klebanoff CA (2004). Bedside to bench and back again: how animal models are guiding the development of new immunotherapies for cancer. *Journal of Leukocyte Biology*.

[21] Liao YP, Wang CC, Schaue D, Iwamoto KS, McBride WH (2009). Local irradiation of murine melanoma affects the development of tumour-specific immunity. *Immunology*.

[22] Lugade AA, Moran JP, Gerber SA, Rose RC, Frelinger JG, Lord EM (2005). Local radiation therapy of B16 melanoma tumors increases the generation of tumor antigen-specific effector cells that traffic to the tumor. *Journal of Immunology*.

[23] Stone HB, Peters LJ, Milas L (1979). Effect of host immune capability on radiocurability and subsequent transplantability of a murine fibrosarcoma. *Journal of the National Cancer Institute*.

[24] Lee Y, Auh SL, Wang Y (2009). Therapeutic effects of ablative radiation on local tumor require CD8^+^ T cells: changing strategies for cancer treatment. *Blood*.

[25] Dewan MZ, Galloway AE, Kawashima N (2009). Fractionated but not single-dose radiotherapy induces an immune-mediated abscopal effect when combined with anti-CTLA-4 antibody. *Clinical Cancer Research*.

[26] Schaue D, Comin-Anduix B, Ribas A (2008). T-cell responses to survivin in cancer patients undergoing radiation therapy. *Clinical Cancer Research*.

[27] Restifo NP, Antony PA, Finkelstein SE (2002). Assumptions of the tumor ‘escape’ hypothesis. *Seminars in Cancer Biology*.

[28] Friedman EJ (2002). Immune modulation by ionizing radiation and its implications for cancer immunotherapy. *Current Pharmaceutical Design*.

[29] Overwijk WW, Theoret MR, Finkelstein SE (2003). Tumor regression and autoimmunity after reversal of a functionally tolerant state of self-reactive CD8^+^ T cells. *Journal of Experimental Medicine*.

[30] Gattinoni L, Finkelstein SE, Klebanoff CA (2005). Removal of homeostatic cytokine sinks by lymphodepletion enhances the efficacy of adoptively transferred tumor-specific CD8^+^ T cells. *Journal of Experimental Medicine*.

[31] Chiang CS, McBride WH (1991). Radiation enhances tumor necrosis factor *α* production by murine brain cells. *Brain Research*.

[32] Quarmby S, Kumar P, Kumar S (1999). Radiation-induced normal tissue injury: role of adhesion molecules in leukocyte-endothelial cell interactions. *International Journal of Cancer*.

[33] Ganss R, Ryschich E, Klar E, Arnold B, Hämmerling GJ (2002). Combination of T-cell therapy and trigger of inflammation induces remodeling of the vasculature and tumor eradication. *Cancer Research*.

[34] Nikitina E, Gabrilovich DI (2001). Combination of *γ*-irradiation and dendritic cell administration induces a potent antitumor response in tumor-bearing mice: approach to treatment of advanced stage cancer. *International Journal of Cancer*.

[35] Ahn GO, Brown JM (2009). Influence of bone marrow-derived hematopoietic cells on the tumor response to radiotherapy: experimental models and clinical perspectives. *Cell Cycle*.

[36] Kachikwu EL, Iwamoto KS, Liao YP Radiation enhances regulatory T cell representation.

[37] McBride WH, Chiang CS, Olson JL (2004). A sense of danger from radiation. *Radiation Research*.

[38] Yee C, Riddell SR, Greenberg PD (2001). In vivo tracking of tumor-specific T cells. *Current Opinion in Immunology*.

[39] Dunn GP, Bruce AT, Ikeda H, Old LJ, Schreiber RD (2002). Cancer immunoediting: from immunosurveillance to tumor escape. *Nature Immunology*.

[40] Howie S, McBride WH (1982). Tumor-specific T helper activity can be abrogated by two distinct suppressor cell mechanisms. *European Journal of Immunology*.

[41] Spiotto MT, Fu YX, Schreiber H (2003). Tumor immunity meets autoimmunity: antigen levels and dendritic cell maturation. *Current Opinion in Immunology*.

[42] Marks LB, Hall EJ, Brenner DJ (1995). Extrapolating hypofractionated radiation schemes from radiosurgery data: regarding Hall et al, IJROBP 21:819–824; 1991 and Hall and Brenner, IJROBP 25:381–385; 1993. *International Journal of Radiation Oncology Biology Physics*.

[43] Wang JZ, Huang Z, Lo SS, Yuh WTC, Mayr NA (2010). A generalized linear-quadratic model for radiosurgery, stereotactic body radiation therapy, and high-dose rate brachytherapy. *Science Translational Medicine*.

[44] Hadziahmetovic M, Loo BW, Timmerman RD (2010). Stereotactic body radiation therapy (stereotactic ablative radiotherapy) for stage I non-small cell lung cancer–updates of radiobiology, techniques, and clinical outcomes. *Discovery medicine*.

[45] Cho J, Kodym R, Seliounine S, Richardson JA, Solberg TD, Story MD (2010). High dose-per-fraction irradiation of limited lung volumes using an image-guided, highly focused irradiator: simulating stereotactic body radiotherapy regimens in a small-animal model. *International Journal of Radiation Oncology Biology Physics*.

[46] Wong J, Armour E, Kazanzides P (2008). High-resolution, small animal radiation research platform with X-ray tomographic guidance capabilities. *International Journal of Radiation Oncology Biology Physics*.

[47] Finkelstein SE, Trotti A, Rao N The Florida Melanoma Trial I: a prospective multi-center phase I/II trial of post operative hypofractionated adjuvant radiotherapy
with concurrent interferon-*α*-2b immunotherapy in the treatment of advanced stage III melanoma with long term toxicity follow up.

[48] Finkelstein SE, Iclozan C, Bui MM Combination of external beam radiotherapy (EBRT) with intratumoral injection of dendritic cells as neo-adjuvant treatment of high-risk soft tissue sarcoma patients.

